# Two scales of distribution and biomass of Antarctic krill (*Euphausia superba*) in the eastern sector of the CCAMLR Division 58.4.2 (55°E to 80°E)

**DOI:** 10.1371/journal.pone.0271078

**Published:** 2022-08-24

**Authors:** Martin J. Cox, Gavin Macaulay, Madeleine J. Brasier, Alicia Burns, Olivia J. Johnson, Rob King, Dale Maschette, Jessica Melvin, Abigail J. R. Smith, Christine K. Weldrick, Simon Wotherspoon, So Kawaguchi

**Affiliations:** 1 Australian Antarctic Division, Kingston, Tasmania, Australia; 2 Australian Antarctic Program Partnership, Institute for Marine and Antarctic Studies, University of Tasmania, Hobart, Tasmania, Australia; 3 Aqualyd Limited, Wakefield, New Zealand; 4 Institute for Marine and Antarctic Studies, University of Tasmania, Hobart, Tasmania, Australia; 5 School of Life and Environmental Sciences, University of Sydney, Sydney, NSW, Australia; 6 Taronga Institute of Science and Learning, Taronga Conservation Society Australia, Mosman, NSW, Australia; MARE – Marine and Environmental Sciences Centre, PORTUGAL

## Abstract

Regular monitoring is an important component of the successful management of pelagic animals of interest to commercial fisheries. Here we provide a biomass estimate for Antarctic krill (*Euphausia superba*) in the eastern sector of the Commission for the Conservation of Antarctic Marine Living Resources (CCAMLR) Division 58.4.2 (55°E to 80°E; area = 775,732 km^2^) using data collected during an acoustic-trawl survey carried out in February and March 2021. Using acoustic data collected in day-time and trawl data, areal biomass density was estimated as 8.3 gm^-2^ giving a total areal krill biomass of 6.48 million tonnes, with a 28.9% coefficient of variation (CV). The inaccessibility of the East Antarctic makes fisheries-independent surveys of Antarctic krill expensive and time consuming, so we also assessed the efficacy of extrapolating smaller surveys to a wider area. During the large-scale survey a smaller scale survey (centre coordinates -66.28°S 63.35°E, area = 4,902 km^2^) was conducted. We examine how representative krill densities from the small-scale (Mawson box) survey were over a latitudinal range by comparing krill densities from the large-scale survey split into latitudinal bands. We found the small scale survey provided a good representation of the statistical distribution of krill densities within its latitudinal band (KS-test, *D* = 0.048, *p*-value = 0.98), as well as mean density (*t*-test *p*-value = 0.44), but not outside of the band. We recommend further *in situ* testing of this approach.

## Introduction

Antarctic krill (*Euphausia superba*, hereafter krill) is a keystone species of the Southern Ocean (SO) ecosystem, being an important grazer, linking the biological pump and facilitating carbon transport from the pelagic to the benthic realms [1], and are a critical prey for baleen whales, seals and seabirds [2].

Commercial fisheries in the region are managed by practices agreed by member nations of the Convention on the Conservation of Antarctic Marine Living Resources (CCAMLR). Management procedures set catch limits and trigger levels for agreed spatial strata, or subareas using conservation measures [3]. Precautionary yield estimates are derived from population parameters in a 20-year projected stock assessment model (the generalised yield model (GYM); [4]). The biomass estimated by acoustic-trawl surveys are then multiplied by the precautionary yield to set catch limits for the krill fishery.

After a hiatus of over two decades, there is renewed interest in fishing for krill in the East Antarctic [5] and the krill fishery recommenced in the Indian Ocean sector in the 2016/17 fishing season. Historical fishing patterns suggest future fishing grounds will likely form to the east of the 50°E meridian in the Indian Ocean sector [3].

CCAMLR Conservation Measures 51–02 and 51–03 govern the catch limit for Division 58.4.1 and 58.4.2 (i.e., Indian Ocean sector), respectively. Current precautionary catch limits and trigger levels in the Indian Ocean sector are set based on information collected through two large-scale surveys conducted by Australia in Divisions 58.4.1 in 1996 (BROKE; [6]) and 58.4.2 in 2006 [7]. Catch limits for both Divisions are further split into two east and west divisions. The most recent krill biomass survey for Division 58.4.1 (80–150°E) was conducted by Japan in 2019 [8], however the entire Division 58.4.2 was last surveyed in 2006 [7].

The Antarctic environment is changing rapidly and these changes are considered to affect the krill population [9]. A changing environment requires increased regular monitoring of krill to ensure precautionary catch limits are based on accurate stock information. However, surveying in the East Antarctic presents logistical challenges. Ship time is expensive (c. 70,000 USD/day in 2022) and the transit time to the East Antarctic is at least eight days from the nearest port in Australia and the subareas are large (this survey 775,732 km^2^; entire 58.4.2 division 1,585,062 km^2^). One possible approach to regular monitoring is to conduct a series of small scale surveys, to ‘fill-in-the-gaps’ (in time) between large-scale surveys. Smaller scale surveys could be conducted as a component of multi-disciplinary science voyages, or as part of annual resupply voyages to Antarctic research stations. Before this approach could be objectively assessed it is important to learn how representative small-scale surveys are of larger-scale patterns in krill density.

The objectives of this research were two-fold. Firstly, to update the large-scale biomass estimate of the eastern sector of the CCAMLR Division 58.4.2 (55°E to 80°E). Secondly, to estimate krill biomass for a smaller-scale survey and compare this estimate to the distribution of krill density found in the main survey within latitudinal boundaries.

## Materials and methods

**Fig 1:**
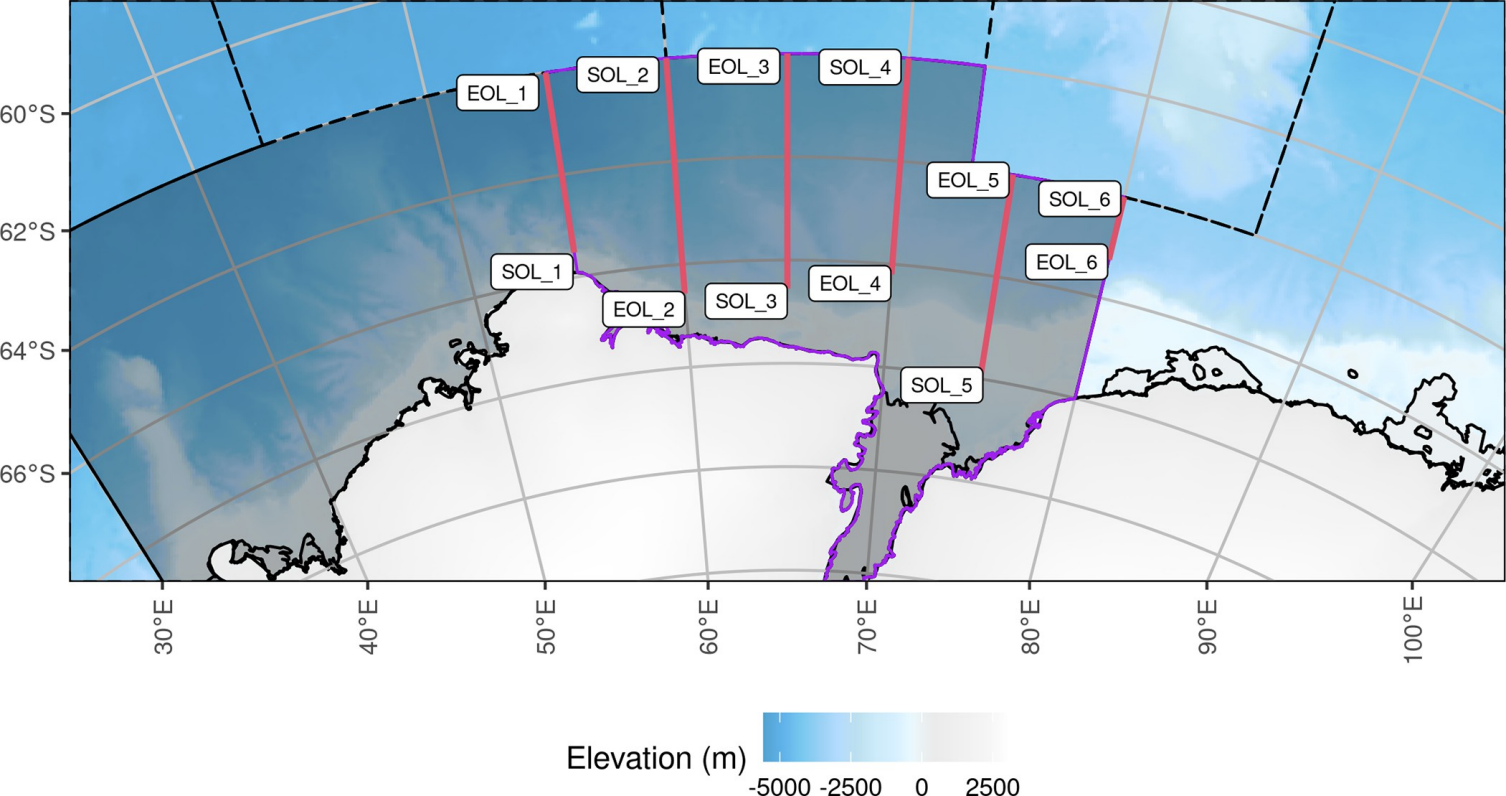
Planned survey transects designed to cover the eastern sector of CCAMLR Division 58.4.2 from 55°E to 80°E (solid border, dark area), with other CCAMLR Division borders shown as dashed lines. The sector of Division 58.4.2 covered by the survey is shown with a purple boundary. The survey transects are solid red lines, labelled with the start-of-line (SOL) and end-of-line (EOL). Acoustic samples (integration intervals) that fall outside of Division 58.4.2, i.e. the northern ends (north of 64°S) of transects 5 and 6, were not used in krill density and biomass estimation. The background bathymetry grid is from The GEBCO_2014 Grid, version 20150318, www.gebco.net.

We conducted a multidisciplinary marine science voyage named Trends in Euphausiids off Mawson, Predators, and Oceanography (TEMPO) from the 94 m long research vessel, RV *Investigator*. The vessel departed Hobart, Australia, on 29 January and returned to Hobart on 24 March 2021, and conducted an acoustic-trawl biomass survey in the eastern sector of the CCAMLR Division 58.4.2 from 13 February to 12 March 2021. The area of operation spanned 62°S to 68°S and 55°E to 80°E (Fig 1). This research was conducted under the Antarctic Marine Living Resources Conservation Act 1981 (Australia) Permit AMLR 20-23-4512.

### Acoustic data collection and calibration

Active acoustic data were collected using a calibrated EK80 scientific echosounder (Simrad, Horten Norway) operating at 18, 38, 70, 120, 200 and 333 kHz. The echosounder transducers were mounted on a drop keel (water depth = 8.5 m). Echosounder cold water calibration took place in open water at the start of the first transect (SOL_1, 65° 31.46’ S 55° 03.01’ E) on 13 February 2021 and followed the procedures of Demer et al. [10] including those for gain estimation (see S1 Table for 120 kHz calibration parameters). Six acoustic transects were designed to cover the eastern sector of CCAMLR Division 58.4.2 (Fig 1) and were identical to the eastern transects of the BROKE-West survey [7, 11].

During the survey, acoustic line transects were observed during day and night at an average vessel speed of 7.3 knots. Transects were divided into segments, where a segment was a continuously observed proportion of a transect bounded by other activities, such as trawling and CTD casts.

### Net sampling

A rectangular mid-water trawl (RMT 8 + 1 [12]) with an 8 m^2^ mouth opening and mesh size of 4.5 mm was used to sample krill and the 1 m^2^ opening and 300 micron mesh size sampled smaller organisms. Trawls were either ‘target-trawls,’ i.e., responsive fishing when krill-like echoes were observed using the vessel’s echo sounders, or routine (i.e., fishing at pre-planned survey stations). Trawls were hauled at 2 knots through water speed and routine trawls sampled the upper 200 m of the water column. The routine trawl net was open for 16 to 18 minutes and the target trawls 7 to 9 minutes.Trawls outside of the survey area (*n* = 4) were excluded from the analysis, as were individual krill with a total length of less than 10 mm.

### Krill wetmass

Individual krill wet-mass (*WW*, g) was weighed using motion compensated Ohaus balances (AX224; Parsippany, USA) and the total length (*TL*, mm) of each weighed krill was measured. The krill length-to-wet mass relationship was modelled using a power law with parameters (*a* and *b*) estimated using nonlinear least squares:

$$WW(TL\vee a,b)=a\times {TL}^{b}$$ (1)

Estimation was carried out using stats::nls() in R version 4.0.5 [13]. Uncertainty was estimated using a non-parametric bootstrap (999 simulations) with trawl as the sampling unit.

### Acoustic data processing

All analyses were carried out on the calibrated 120 kHz data. The 120 kHz data were processed to remove surface (0 to 15 m water depth) and seabed returns, as well as background and intermittent noise (see Fig 7 in [14] the effect of varying the noise removal filter *S*_*v*_ threshold). The TEMPO voyage 120 kHz mean volume backscattering strength (S_*v*_, [15]) data was processed with a noise removal threshold of -40 dB re 1 m^-1^ (solid black line; S1 Fig) which did not remove any krill echoes. Lower value thresholds (-44 to -41 dB re 1 m^-1^) removed krill echoes (S1 Fig for the selection of *S*_*v*_ noise threshold). Acoustic data processing was carried out using Echoview software (version 11.1, Echoview, Hobart, Australia), automated via COM-objects using EchoviewR (version 1.1.18; [16]). Sound speed and absorption coefficients were applied to each transect segment and were calculated from the nearest voyage CTD station using the harmonic mean of sound speed calculated from CTD samples between 10 m and 250 m water depth.

Krill echoes were delineated in the 120 kHz data using the CCAMLR-endorsed ‘swarms-based’ method [5, 14].

### Krill target strength

Krill target strength values from the 2019 large-scale survey [14] were used to scale Nautical Area Scattering Coefficient (NASC, units: m^2^ nautical mile ^-2^) to areal biomass density (units gm^-2^). Krafft et al. [14] target strength values were calculated using the MATLAB code of [17] which was an update of [18]. The 120 kHz length-specific krill target strengths were downloaded from: https://github.com/ccamlr/2019Area48Survey/blob/master/results/SDWBA-TS-38-120-200.csv on 2021-05-17.

### Areal biomass density

Krill echoes were integrated in one nautical mile by 250 m deep intervals (integration interval hereafter). Areal krill biomass density was calculated by multiplying the linear measure of acoustic energy, *s*_*A*_, using a conversion factor *C* defined as:

$$C=\frac{\sum f_{i}WW(l_{i})}{\sum f_{i}\sigma_{sp}(l_{i})}\times \frac{1}{1852^{2}}$$ (2)

where *σ*_*sp*_ is the linear form of target strength *σ*_*sp*_ = 4*π*10^{*TS*/10}^ (with units of m^2^) and *f*_*i*_ is the number of krill in the *i*th length class of length *l*_*i*_.

### Mean areal krill biomass density

Mean areal krill biomass density (herein krill density; *ρ*, gm^-2^) and associated sampling variance, *Var[ρ]*, was estimated using the random sampling theory estimator of Jolly and Hampton [19]. Transects on the survey area boundary (transects one and six; Fig 1) were downweighted by 0.5 to account for half the effective transect coverage area falling outside the survey boundary.

### Day-night krill density differences

After [20], day and night were defined by civil day-night, calculated using the function *getSunlightTimes()* in the *R* package *suncalc* ([21]; version 0.5.0). The date/time and position at the start of each integration interval was used to categorise the interval into day or night. Differences in krill density were examined by: (i) comparing krill density integrated at one nautical mile intervals; (ii) using a LOESS smoother to look for evidence of diel vertical migration in krill swarm depth, weighted by volumetric density (higher the density, greater the weight).

### Deviation from planned transect

A correction was applied to account for vessel deviation from the planned transects. Following Hewitt et al. [22], the expected change in latitude for each nautical mile surveyed of planned transect *ΔL* was calculated for each transect. The latitude made good, i.e., actually surveyed, *ΔL*, was calculated as the difference between the observed latitudes at the beginning and end of each one nautical mile integration interval. The weighting for the *i*th integration interval was calculated as:

$$W_{Ii}=\frac{\left|\Delta L|-|\left(\Delta L-\Delta \hat{L}\right)\right|}{\left|\Delta L\right|}$$ (3)

The interval weighting, *W*_*I*_, was only applied in situations where deviation from the planned transect exceeded 10%, i.e., *W*_*I*_*<0*.*9*. Where deviation was less than 10%, the interval weight remains as *W*_*I*_ = 1, giving:

$$W_{Ii}=\left\{ \begin{array}{c} W_{Ii}\geq 0.9,1\\ W_{Ii}<0.9,W_{Ii} \end{array}\right.$$ (4)

After (Eq 8, [22]) the weight of a transect will be reduced where one or more integration intervals have *W*_*I*_*<0*.*9* deviation:

$$L_{j}=\sum_{i=1}^{N_{j}}W_{I,i}$$ (5)

where, *L*_*j*_ is the length of the *j*th transect and *N*_*j*_ is the number of integration intervals in the *j*th transect. Each of the transect lengths *L* are then used in the random sampling theory estimator ([19], Eqs 9 to 13 in (Hewitt et al. [22], when there is a single stratum, as is the case here.

### Latitudinal variation

To investigate whether the small scale ‘Mawson Box’ survey was representative of a larger area, we divided the integration intervals from the large-scale survey into latitudinal bands, with the latitudinal height of each band equal to the latitudinal extent of the Mawson Box survey. We compared the presence-absence of krill and conditional density (*ρ[p>0]*) in integration intervals between the Mawson Box and each of the latitudinal bands.

## Results

The acoustic-trawl survey of krill consisted of six line transects observed between 13 February 2021 and 10 March 2021. The total transect distance run during the acoustic survey was 1,188 nautical miles and included 34 net trawls.

### Acoustic transects

Large scale transect length varied from 72 nautical miles to 297 nautical miles (Table 1). Transects one and six were run along the western and eastern boundaries respectively of the eastern sector of CCAMLR Division 58.4.2 (Fig 1) so data from these transects were down weighted by 0.5 in the biomass estimation procedure.

**Table 1:** Acoustic transect duration (times in UTC), length and coverage weighting of the large scale survey.

Transect	Start date/time	End date/time	Duration (hours)	Length (nautical miles)	Coverage weighting
1	2021-02-13 15:13	2021-02-15 19:45	53	204	0.5
2	2021-02-16 16:38	2021-02-20 22:11	102	297	1.0
3	2021-02-23 15:18	2021-02-26 22:17	79	276	1.0
4	2021-02-27 21:25	2021-03-03 17:49	92	251	1.0
5	2021-03-05 04:17	2021-03-08 06:11	74	245	1.0
6	2021-03-10 19:58	2021-03-11 19:00	23	72	0.5

### Net sampling

**Fig 2:**
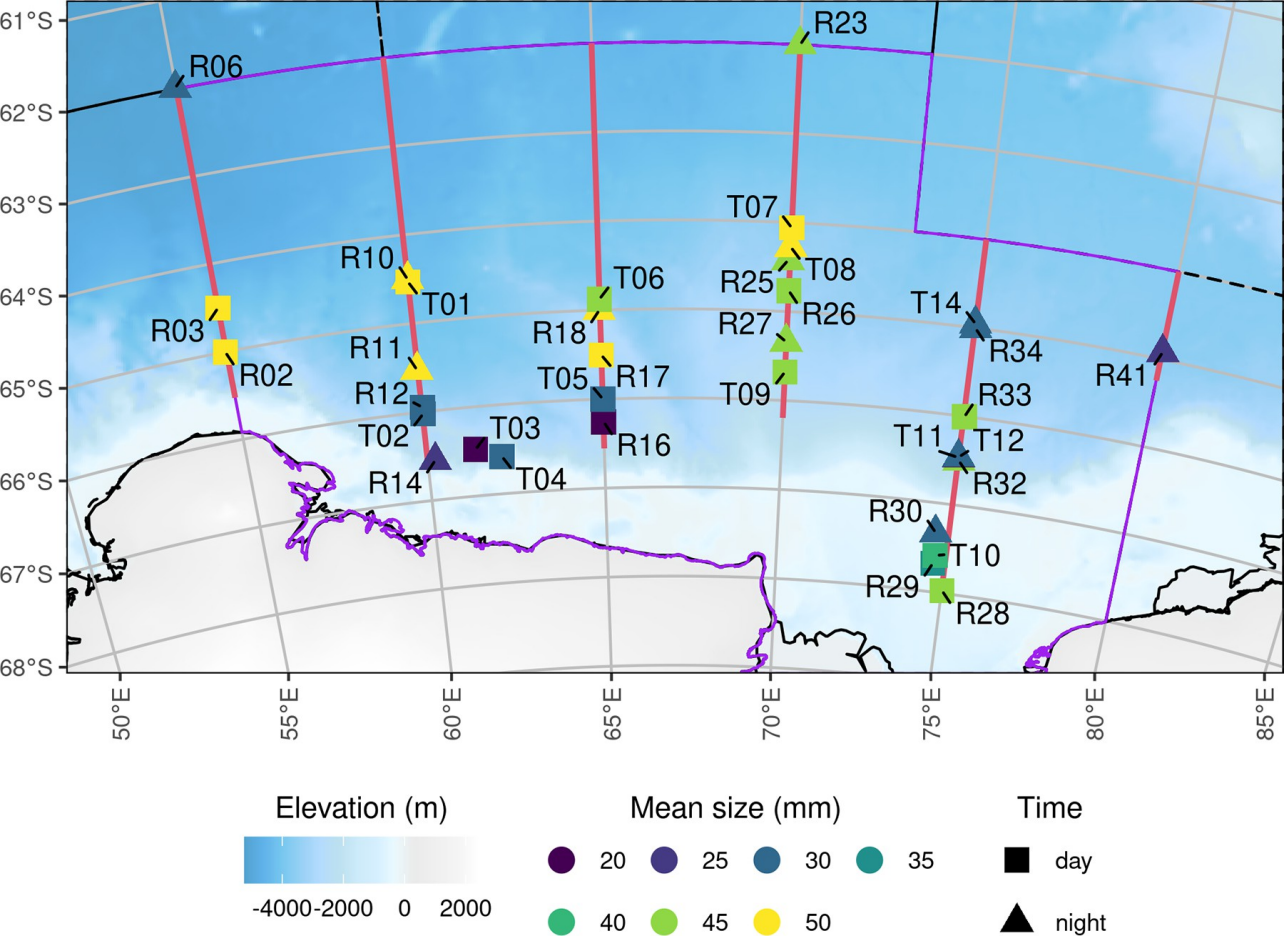
Net trawl locations where krill were sampled. Trawl type: routine or target are denoted by R and T respectively in the station identification code. Day and night sampling are denoted by square or triangle symbols and colour is the mean length of krill in trawl. The background bathymetry grid is from The GEBCO_2014 Grid, version 20150318, www.gebco.net.

Of the 34 trawls, 21 were routine (planned) trawls and 13 were target (responsive) trawls (Fig 2). After Lawson et al. [23] and references therein, we examined the proportion of catch that arose from aggregating species, in this case Antarctic krill and ice krill (*Euphausia crystallorophias*). Across all nets, the ratio of Antarctic krill to ice krill was 7.4 to 1.

### Krill length frequency distribution

**Fig 3:**
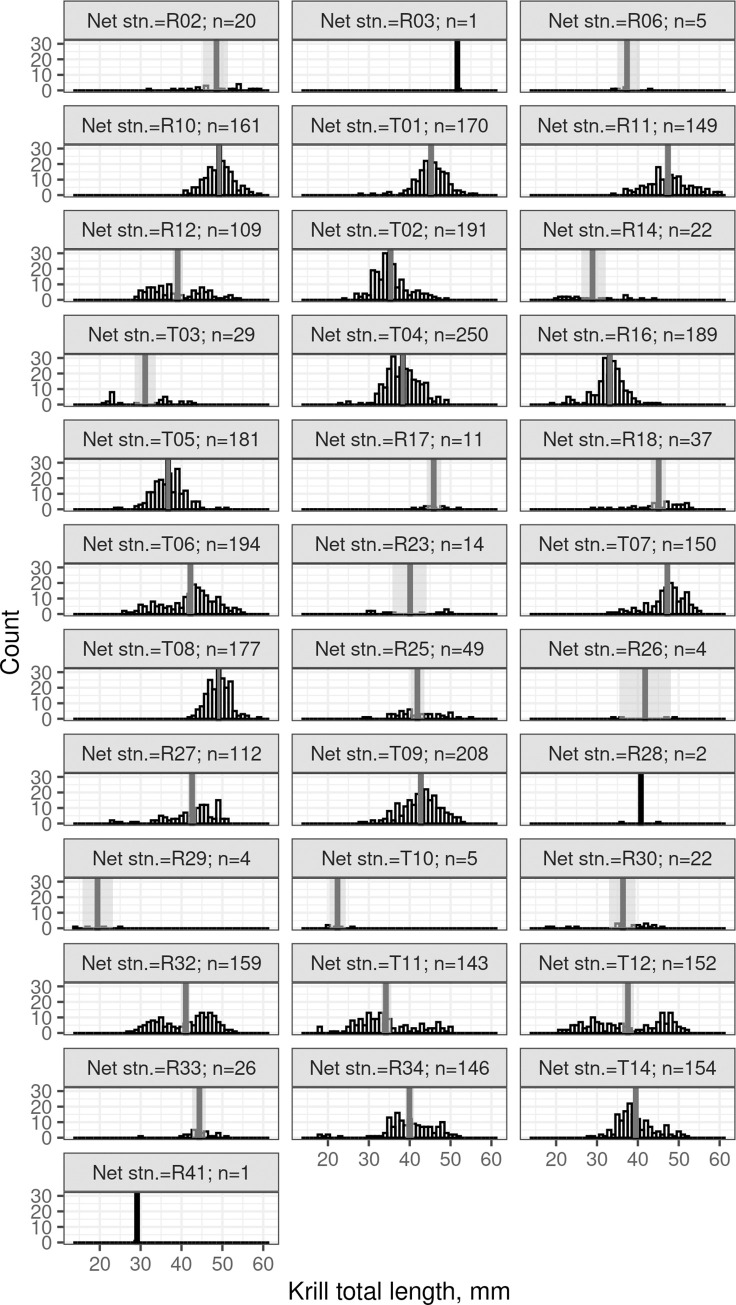
Routine (planned) and target (responsive) net trawls. Krill total length (mm) here are from the rectangular mid-water trawl (RMT) samples. The mean TL for each trawl is a solid vertical line and for nets with 30 or more samples, the 95% confidence interval of the mean TL is shown as a vertical grey region.

There were no obvious patterns in krill length frequency distributions by net trawl, although there was variation in trawl mean length and length distribution (Figs 2 & 3).

**Fig 4:**
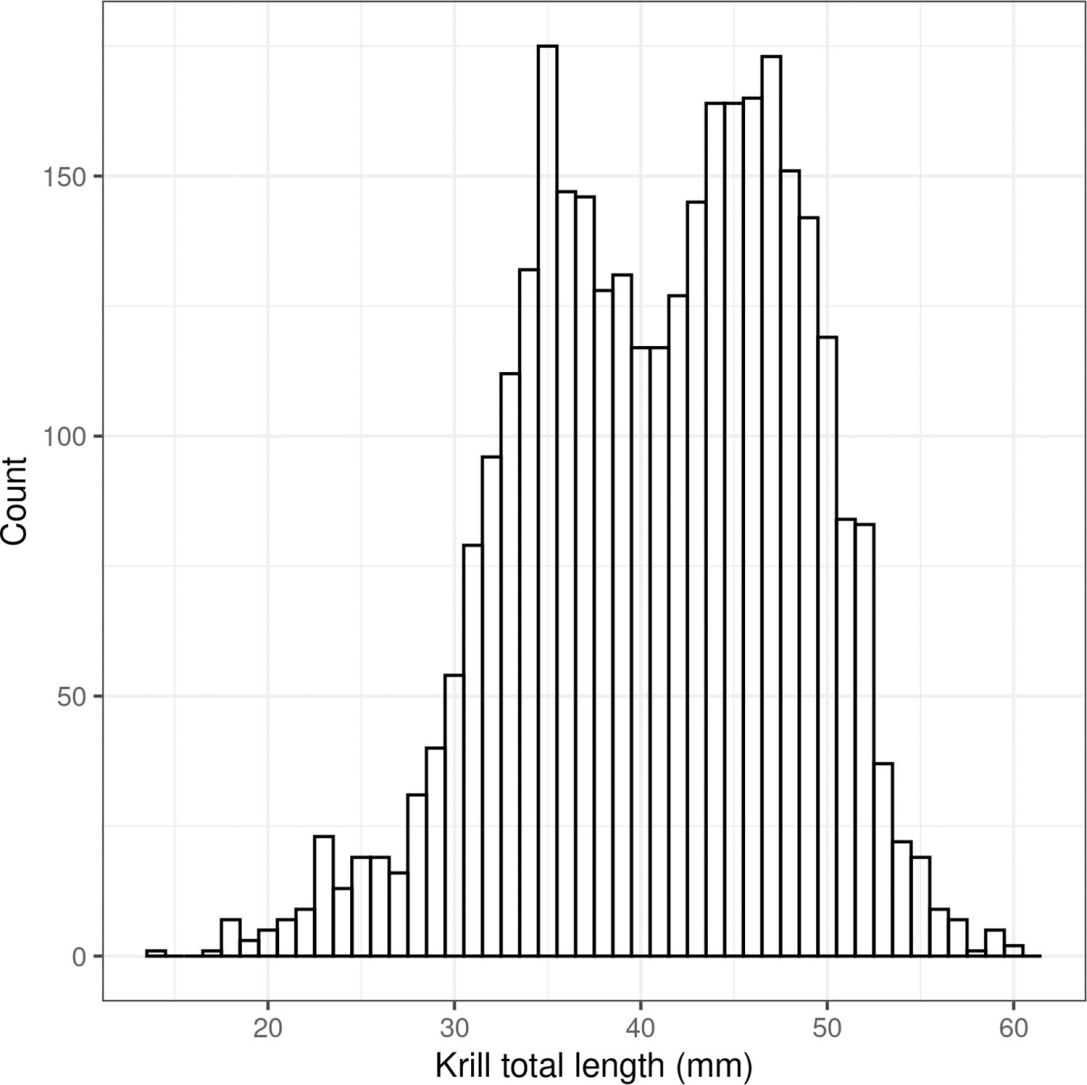
Length frequency distribution of krill combined from all trawls (n = 3,247) and used to calculate the NASC to areal krill biomass density.

Given there was insufficient coverage to discern spatial or temporal patterns in krill mean total length or length frequency distribution for each trawl, the krill length frequency distributions were combined into a single distribution (Fig 4) when calculating the NASC to areal biomass conversion factor (*C*; *Eq* 1).

### Krill length to wetmass

**Fig 5:**
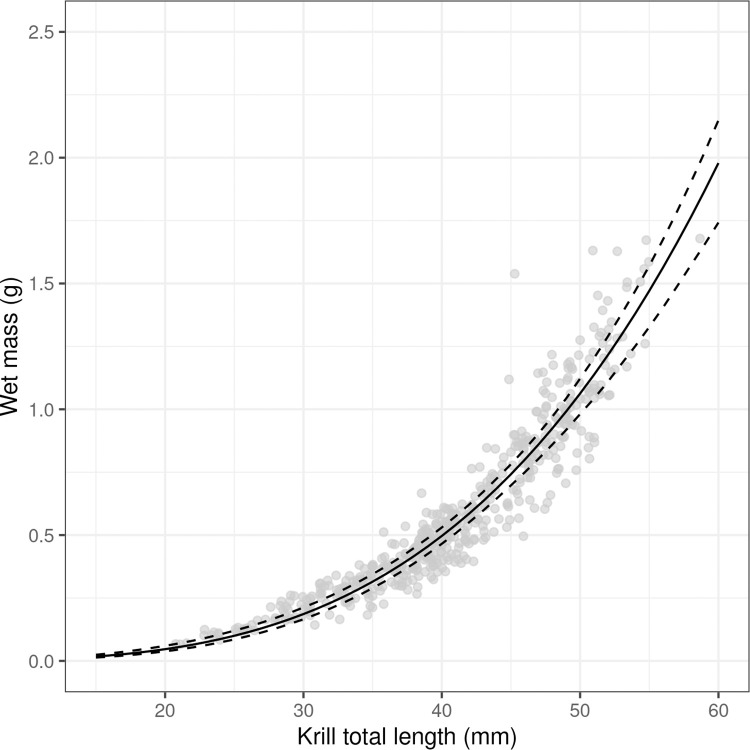
Krill total length to wet mass relationship. Observations are grey semi-transparent dots, the fitted model is a solid black line and the 95% confidence intervals, determined by non-parametric bootstrap, are dashed lines.

Krill total length to wet mass was modelled as a power function using data from individual krill (*n* = 502). The estimated parameters were $\hat{a}$ = 1.71 x 1e-6 (CV = 22.8%) and $\hat{b}$ = 3.41 (CV = 1.7%) that produced the solid curved line in Fig 5.

### Krill target strength and areal biomass density

**Fig 6:**
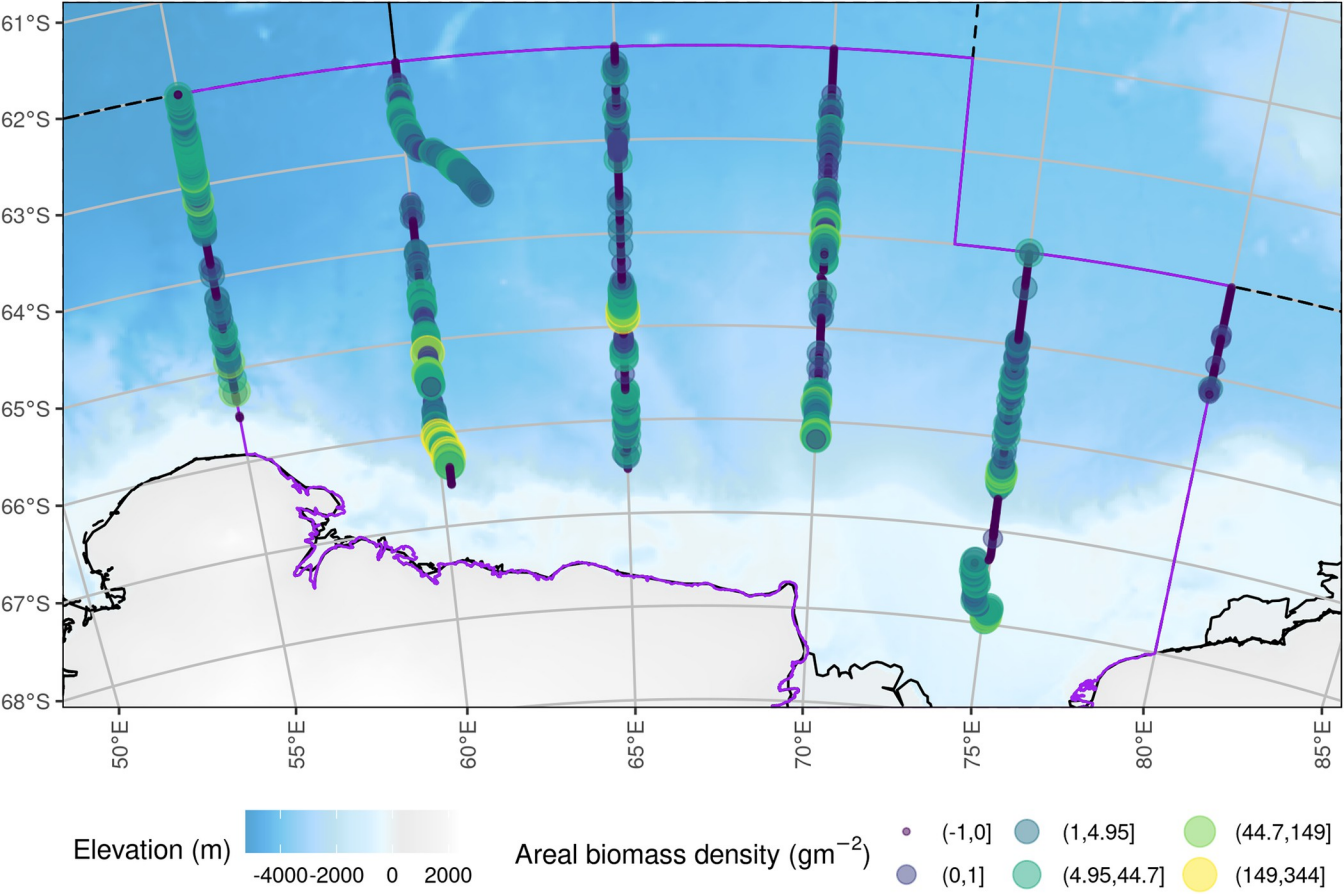
Krill areal biomass density per one nautical mile integration interval to a 250 m water depth. Circle colour is related to a category of krill areal biomass density denoted by the intervals in the legend. The eastern sector of CCAMLR Division 58.4.2 covered by the survey is shown as a grey shaded area with a purple boundary. The background bathymetry grid is from The GEBCO_2014 Grid, version 20150318, www.gebco.net.

The 120 kHz NASC to krill biomass density conversion factor was *C* = 0.348 g m^-2^ n.mil^-2^. There were no obvious spatial patterns in krill biomass density (Fig 6).

### Biomass estimates

Estimated mean areal biomass density, $\hat{\rho }$, from the day and night data was 6.2 gm^-2^, and a total biomass of $\hat{\beta }$ = 4.8 million tonnes of krill (CV = 24.2%) over the 775,732 km^2^ survey area. Transect-by-transect results show the effect of accounting for variation in transect length using transect weights (Table 2) as per [19].

**Table 2:** Day and night data transect level results for the random sampling theory estimator. NB transects one and six are down weighted to account for the transect being placed on the Division eastern and western boundaries respectively (shown in the transect length weight). The transect weight column is the random sampling theory estimator transect weights (based on the reduced transect lengths). Surveying was carried out both day and night.

Transect number	Transect length (nautical mile)	Transect length weight	Transect weight	Biomass density (gm^-2^)	Weighted biomass density (gm^-2^)	Density deviation
1	205.8	0.5	0.519	5.1	2.6	1.25
2	278.7	1.0	1.407	11.4	16.0	26.80
3	275.4	1.0	1.390	5.4	7.5	0.59
4	252.2	1.0	1.273	5.0	6.3	1.45
5	243.3	1.0	1.228	3.7	4.6	5.93
6	72.0	0.5	0.182	0.1	0.0	37.23

The statistical distributions of integration interval-based areal biomass density varied between day and night (KS-test *D* = 0.3, *p*-value = <2e-16). There were also statistically significant (and practical) differences in the mean areal biomass density between day and night (t-test, *t* = 5.33; *p*-value = 1.2e-07) with the 95% confidence intervals for the mean difference between day and night: 3.01 to 6.51 gm^-2^, i.e, day time density was higher.

**Fig 7:**
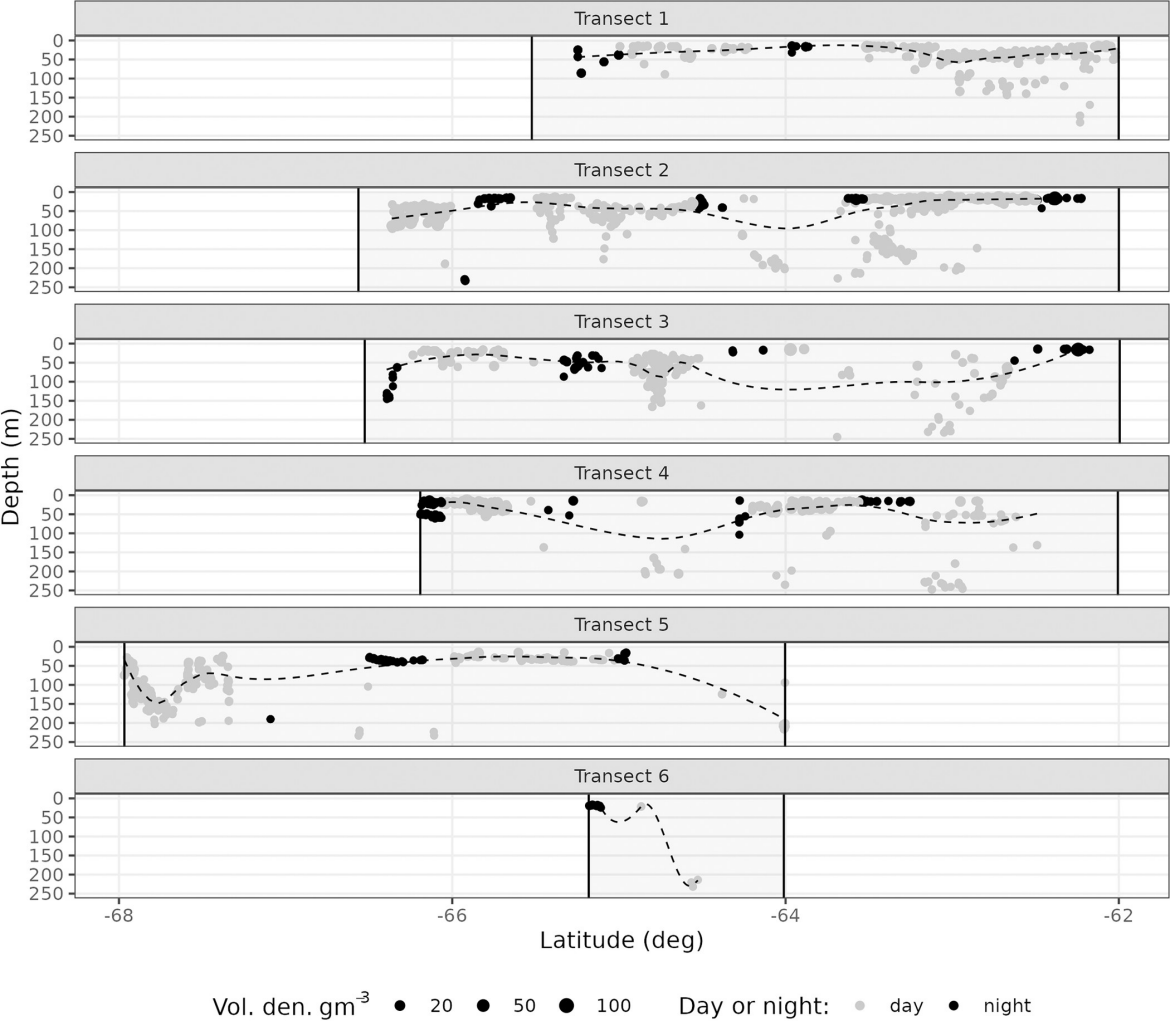
Diel patterns of krill swarm depth and internal density for each transect. Each of the six transects is shown on a separate panel labelled 1 to 6. NB the different start and end latitudes (x-axis) for each transect are denoted as grey rectangles. Day and night are defined as civil day-night and swarms coloured light grey for day and dark grey for night. The area of each swarm circle is proportional to internal swarm volumetric density (gm^-3^) and the black dashed line is a LOESS smooth (span = 0.4) through the centre of mass, i.e. the mean swarm depth weighted by mean swarm internal density.

**Fig 8:**
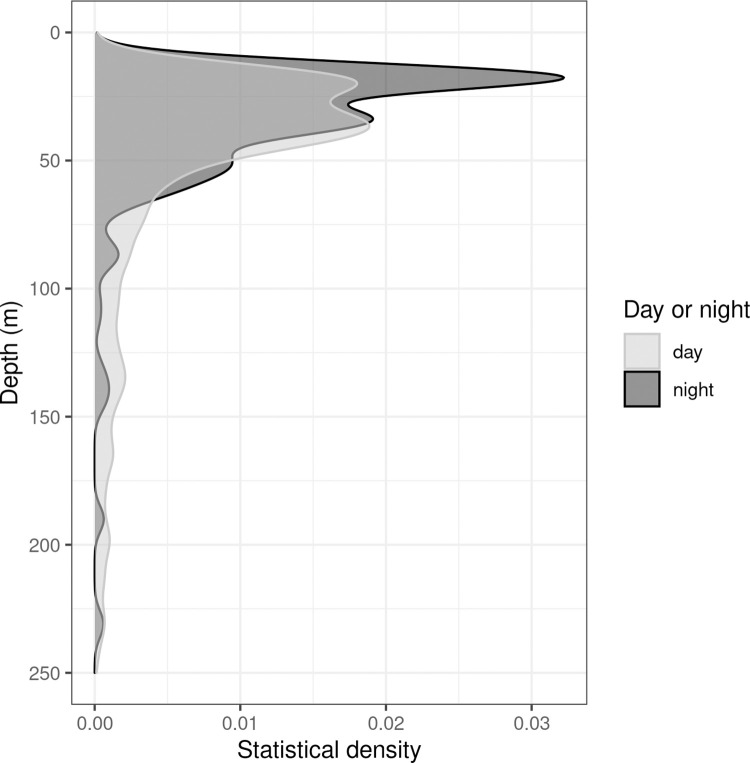
Distribution of krill swarms by water depth, split by day and night.

Analysing swarm depth distribution offers a higher depth resolution than considering echo integration intervals where depth is collapsed. Swarms were found at all sampled depths (Fig 7) during night although typically fewer swarms were found in deeper waters (>100 m). No obvious diurnal pattern emerges examining the loess smooth of swarm depth (weighted by swarm interval density). Also there was no evidence of abrupt changes in krill swarm depth in the water column at the day to night transition (Fig 7). Throughout the survey, however, krill swarm depth distribution varied between day and night (KS-test, *D* = 0.3; *p* < 2e-16), with krill being found closer to the surface at night (peak in krill depth distribution between 15 and 30 m; Fig 8). Also, the krill swarm encounter rate was higher and more variable in the day at 3.7 swarms/nautical mile (CV = 55.8%) compared to the night time encounter rate 1 swarm/nautical mile (CV = 37.7%).

### Recalculation of krill biomass for day-night differences

**Fig 9:**
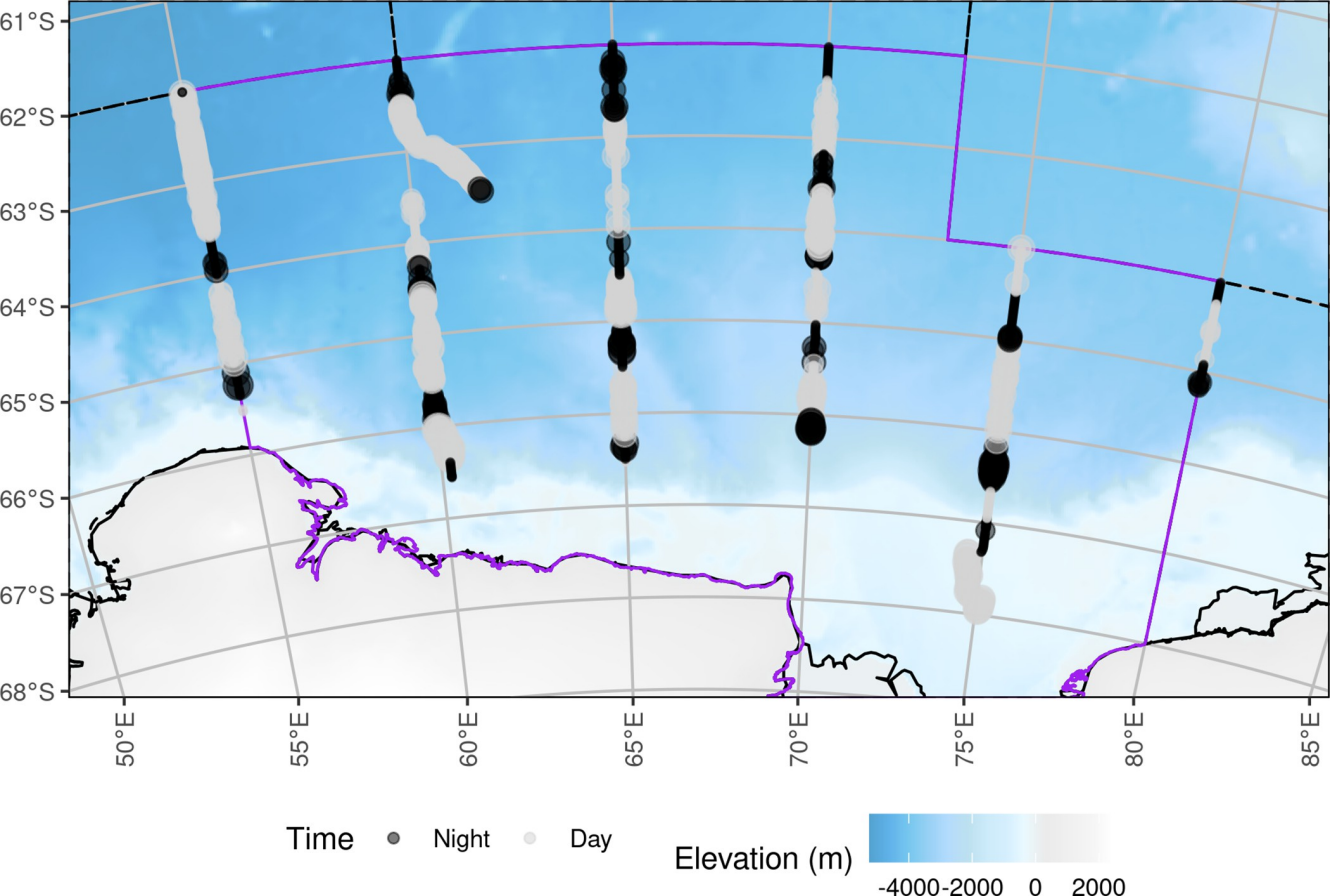
Day/night effort distribution of survey. Point area is proportional to krill areal biomass density. The background bathymetry grid is from The GEBCO_2014 Grid, version 20150318, www.gebco.net.

After finding statistically different day-night differences in krill mean areal biomass density and krill swarm depth distribution we recalculated krill biomass using only the day time data (*n* = 932; 65% of the integration intervals; Fig 9 & Table 3).

**Table 3:** Krill biomass estimate for the eastern sector of the CCAMLR Division 58.4.2.

			Biomass (million tonnes)		
Data name	Density estimate	CV	Estimate	Lower bound	Upper bound
All data	6.2 gm^-2^	24.2%	2.5	4.8	7.1
Day time only	8.3 gm^-2^	28.9%	2.7	6.2	9.7

### Latitudinal variation

**Fig 10:**
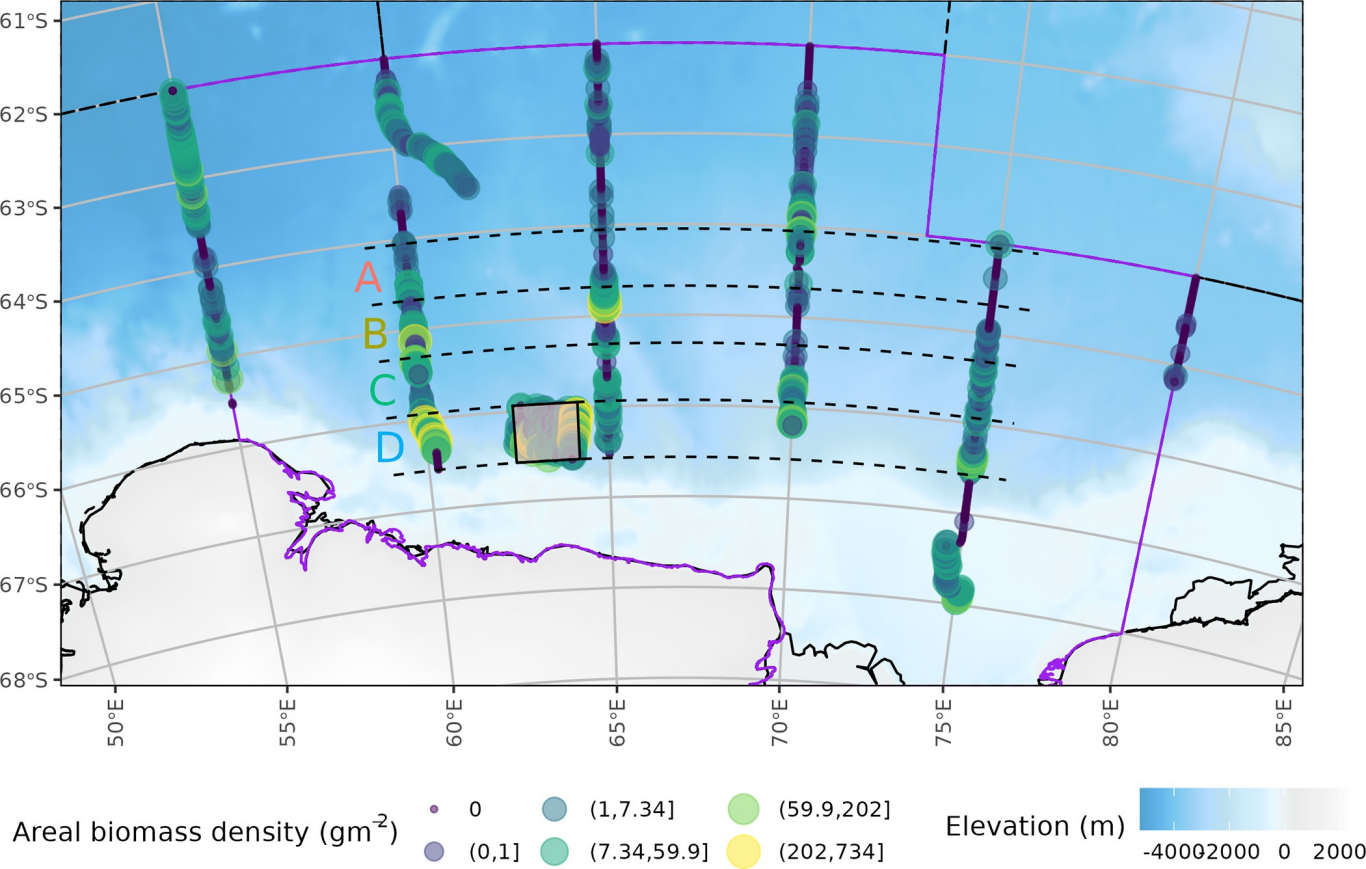
Variation in krill density with latitude. The main-survey transects were divided into four latitudinal bands (black dashed lines; labelled A to D). Latitudinal band width was 0.63° which is equal to the latitudinal width of the Mawson box (shaded rectangle) located in latitudinal band D. NB latitudinal band letters are coloured coded to match Fig 11. The background bathymetry grid is from The GEBCO_2014 Grid, version 20150318, www.gebco.net.

The latitudinal height of the ‘Mawson Box’ survey (purple shaded area Fig 10) was 0.63° (69.9 km). We used this latitudinal height to divide transects 2 to 5 into four equal width bands (dashed lines labelled A to D, Fig 10). Latitudinal band D aligned with the Mawson box enabling comparison with the main survey.

**Fig 11:**
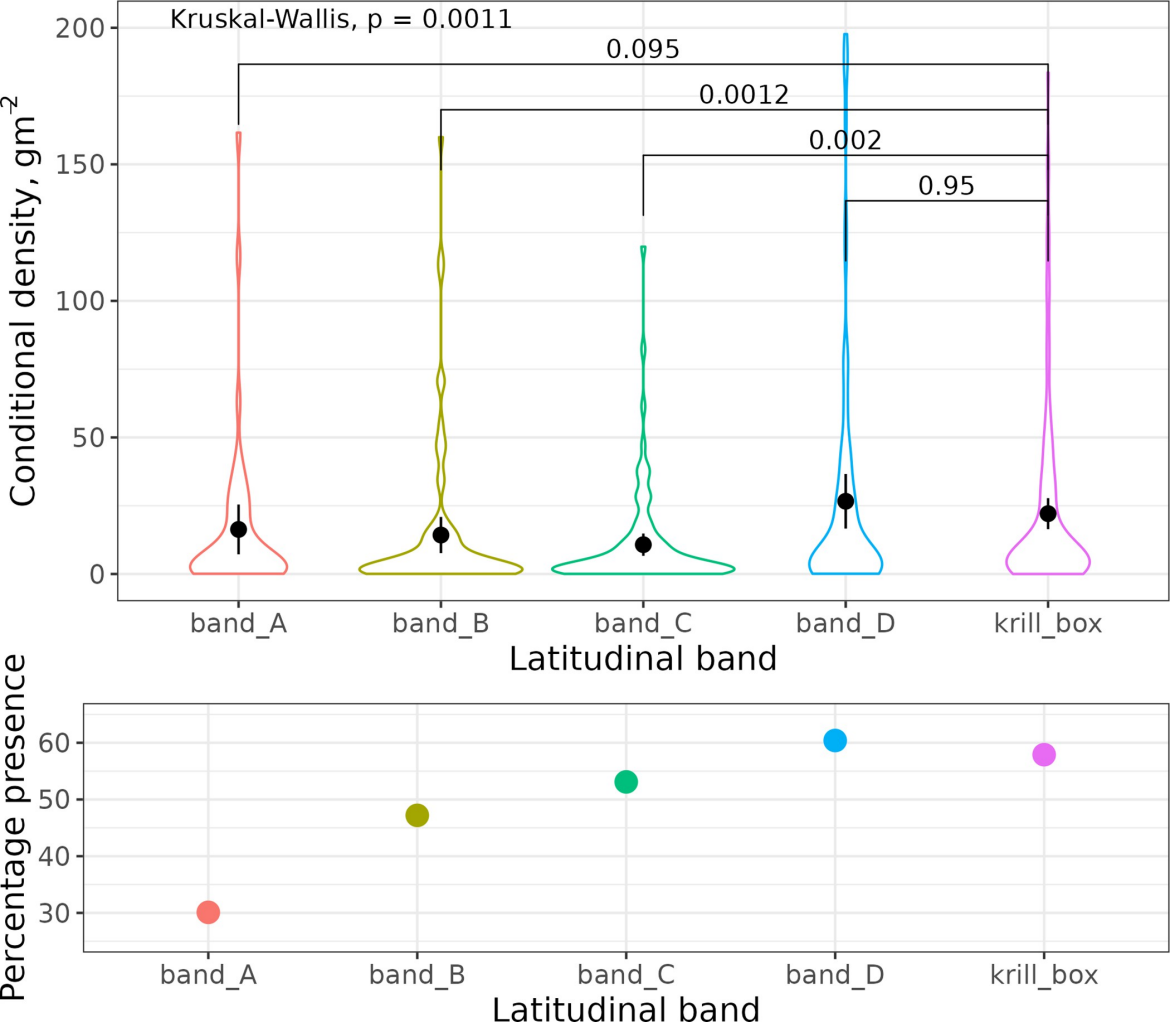
Krill density split into latitudinal bands (see Fig 10 for bands). Krill densities from one nautical mile echo integration intervals from the main survey data were allocated to a band. Mawson box densities are given in the final column. The upper panel is conditional krill density (i.e. all non-zero krill densities; ρ[ρ>0]). The lower panel is the percentage of echo integration intervals containing krill. The solid horizontal lines are p-values from paired t-tests comparing krill densities in each of the bands to krill densities in the Mawson box.

There were significant differences between the conditional krill density in one or more latitudinal bands (Kruskal-Wallis, p = 0.0011; upper panel Fig 11). Pairwise comparisons revealed differences between the Mawson box conditional densities and those in band-B and band-C. Conditional densities in the Mawson box were similar in latitudinal band-D, which is the same latitudinal band as the Mawson box. Interestingly, band-A, the band furthest from the Mawson box (upper panel Fig 11) had similar conditional krill densities as the Mawson box. In contrast, band A had the fewest integration intervals containing krill (lower panel Fig 11). The distribution of krill densities was similar in the Mawson box and band-D (KS-test, D = 0.048, p-value = 0.98) and similar means (t-test, t = -0.78, p-value = 0.44).

## Discussion

From this work, krill biomass for the eastern sector of the CCAMLR Division 58.4.2 is estimated at 4.8 million tonnes (2.5–7.1 million tonnes, lower and upper bounds). Areal biomass density estimated here, (6.4 gm^-2^, CV = 28.9%), is considerably lower than other estimates from East Antarctica (although see [24]). A recalculation of the 2006 BROKE-West (30°E to 80°E; entire CCAMLR Division 58.4.2) krill biomass estimate, revising the work of Jarvis et al. [11], to account for changes in the parameterisation of the krill target strength (TS) model and acoustic data processing gave $\hat{\rho }$ = 20.5 gm^-2^ (CV = 16%) [25]. In March 1996 the adjacent CCAMLR Division (58.4.1; 80° to 150°E) had a krill biomass density of 5.5 gm^-2^ (CV = 17%) [26], however this was calculated with a different target strength model [27] which probably gave a lower biomass density estimate [18] compared to the current TS model [17].

### Diel vertical migration signal

Our analyses found significant differences in krill areal density between day and night. We assessed diel vertical migration (DVM) at three analysis scales: (i) areal biomass density at the one nautical mile integration interval; (ii) evidence of DVM at the scale of swarms, and (iii) the vertical distribution of krill (survey scale). Using method one we found lower areal biomass density during nighttime. The results of method two were equivocal. No consistent changes were found in the vertical distribution of krill swarms at dawn or dusk. This might be due to a change in tilt angle of krill during ascent resulting in a reduction in backscattering cross section, which in turn reduces krill target strength and so the detectability of krill [28]. Also, the low swarm encounter rate makes detecting this signal difficult. The DVM signal is much easier to detect in acoustic scattering layers because the layers are often present as strong echoes (e.g. fish with swim bladders) and are also continuous features, although rarely at high latitudes [29].

For ship-based surveys, the surface zone, in this case from the sea surface to 20 m water depth is unsampled. The peak in night time swarm depth distribution occurred at 30 m water depth, providing some evidence that krill swarms were missed during night time surveys (Fig 8).

Given the day-night differences we found in the statistical distribution of krill areal biomass density and the vertical distribution of krill swarms, we recalculated the biomass estimate using day time data only, which gave a higher biomass estimate and CV compared to combined day and night data.

### Krill morphology and species identification

Seasonal changes in krill morphology will also affect krill target strength (TS; [28, 30]). This survey took 26 days to complete and krill growth during this time will potentially increase krill length and girth [31, 32]. The current krill TS model implements girth as the fatness coefficient [17]. Feeding may change krill material properties (density and sound speed contrasts, also implemented in the current krill TS model; [17]), although it is unclear if seasonal changes or natural variation would dominate TS variance at the spatial scale or over the duration of the survey.

There does not appear to be any systematic spatial pattern in length frequency during survey (Figs 2 & 3). As growth rate of krill in this region is known to range -0.006 to 0.091 mm per day in February and March [33], the entire krill length frequency (Fig 4) was used to convert *s*_*A*_ to *ρ*, both of which eliminate any temporal bias in length frequency data.

Net sampling during the survey was a combination of routine and target trawls. The lower encounter rate of swarms and bad weather days meant that the planned number of target and routine trawls was not reached. Arguably the spatial distribution of krill length frequency was under sampled on this survey, which is why we have not explicitly accounted for spatial variation in krill length frequency (Fig 4).

Ice krill was present in the net samples. The current acoustic sampling and processing methods cannot discriminate between Antarctic krill and ice krill [23] so it is possible that some of the acoustic signal allocated to Antarctic krill, and hence the biomass estimate may actually be from ice krill. Although, this possible confusion between acoustic echoes from ice krill or Antarctic krill will only probably occur in areas of the survey that sample coastal regions [34].

### Latitudinal variation

The overlap in conditional density krill distributions between the Mawson box and latitudinal band D (Figs 10 & 11), coupled with similar krill presence-absence suggests that the Mawson box can be representative of krill distribution within the latitudinal extents of the Mawson box. This suggests that it may be viable to estimate krill density over a large area using a series of small-surveys extrapolated to several, potentially overlapping, latitudinal bands.

The increase in conditional density of krill at the northernmost survey extent (band A, Figs 10 & 11) was possibly caused by a non-linear relationship between krill and its environment (e.g. [35, 36]). It may however, also be caused by individual krill swarms attempting to maintain an internal biomass, despite reduced regional, in this case latitudinal band, biomass [37].

### Summary

Krill biomass for the eastern sector of CCAMLR Division 58.4.2 is estimated at 4.2 million tonnes with areal biomass densities of 6.4 gm^-2^. Observed krill densities were higher in the day than night. This is indicative of krill moving closer to the surface at night above the minimum observation depth of the echosounder. This diel vertical migration pattern is not ubiquitous. Krafft *et al*. [14] for example, did not detect a DVM signal in krill densities during their large-scale survey of the Southwest Atlantic sector of the Southern Ocean.

In order to make the scaling of small surveys to larger areas useful for krill management, i.e. setting krill catch limits, there is more work to be done in variance estimation and more data collection and testing of how representative the small surveys area across latitudinal bands.

## Supporting information

S1 Table Calibration parameters and operation settings for the 120 kHz Simrad EK80 echosounder. (DOCX)

S1 Fig Effect of mean volume backscattering strength (S_v_) noise filter setting on cumulative NASC. The noise removal filter S_v_ threshold can remove low-volume, high-density, krill echoes. Following the procedure of [14] the effect of varying the noise removal filter S_v_ threshold was investigated. The TEMPO voyage S_v_ data was processed with a noise removal threshold of—40 dB re 1 m^-1^ (solid black line) which did not remove any krill echoes. Lower value thresholds (-41 to -44 dB re 1 m^-1^) removed krill echoes.
(TIF)
