# Supplementary material for: Two scales of distribution and biomass of Antarctic krill (Euphausia superba) in the eastern sector of the CCAMLR Division 58.4.2 (55°E to 80°E)
Source: PLoS One. 2022 Aug 24;17(8):e0271078. doi: 10.1371/journal.pone.0271078 (PMC9401115; doi:10.1371/journal.pone.0271078)
Supplement: S1 Table — (DOCX) [file pone.0271078.s001.docx]

## **Table S1**: Calibration parameters and operation settings for the 120 kHz Simrad EK80 echosounder.

| Parameter | Value |
| --- | --- |
| Gain, dB re 1 | 26.28 |
| s_A_ correction, dB re 1 | -0.05 |
| Major axis 3 dB beam angle, ° | 6.63 |
| Major axis angle offset, ° | 0.01 |
| Major axis angle sensitivity | 23.0 |
| Minor axis 3 dB beam angle, ° | 6.65 |
| Minor axis angle offset, ° | -0.07 |
| Minor axis angle sensitivity | 23.0 |
| Sound speed, ms^-1^ | 1442.39 |
| Absorption coefficient, dB m^-1^ | 0.0277 |
| Transmit power, W | 250 |
| Transmit pulse duration, ms | 1.024 |
| Two-way beam angle, dB re 1 sr | -20.70 |
| RMS, dB | 0.19 |
