# Supplementary figures and images for: Two scales of distribution and biomass of Antarctic krill (Euphausia superba) in the eastern sector of the CCAMLR Division 58.4.2 (55°E to 80°E)
Source: PLoS One. 2022 Aug 24;17(8):e0271078. doi: 10.1371/journal.pone.0271078 (PMC9401115; doi:10.1371/journal.pone.0271078)

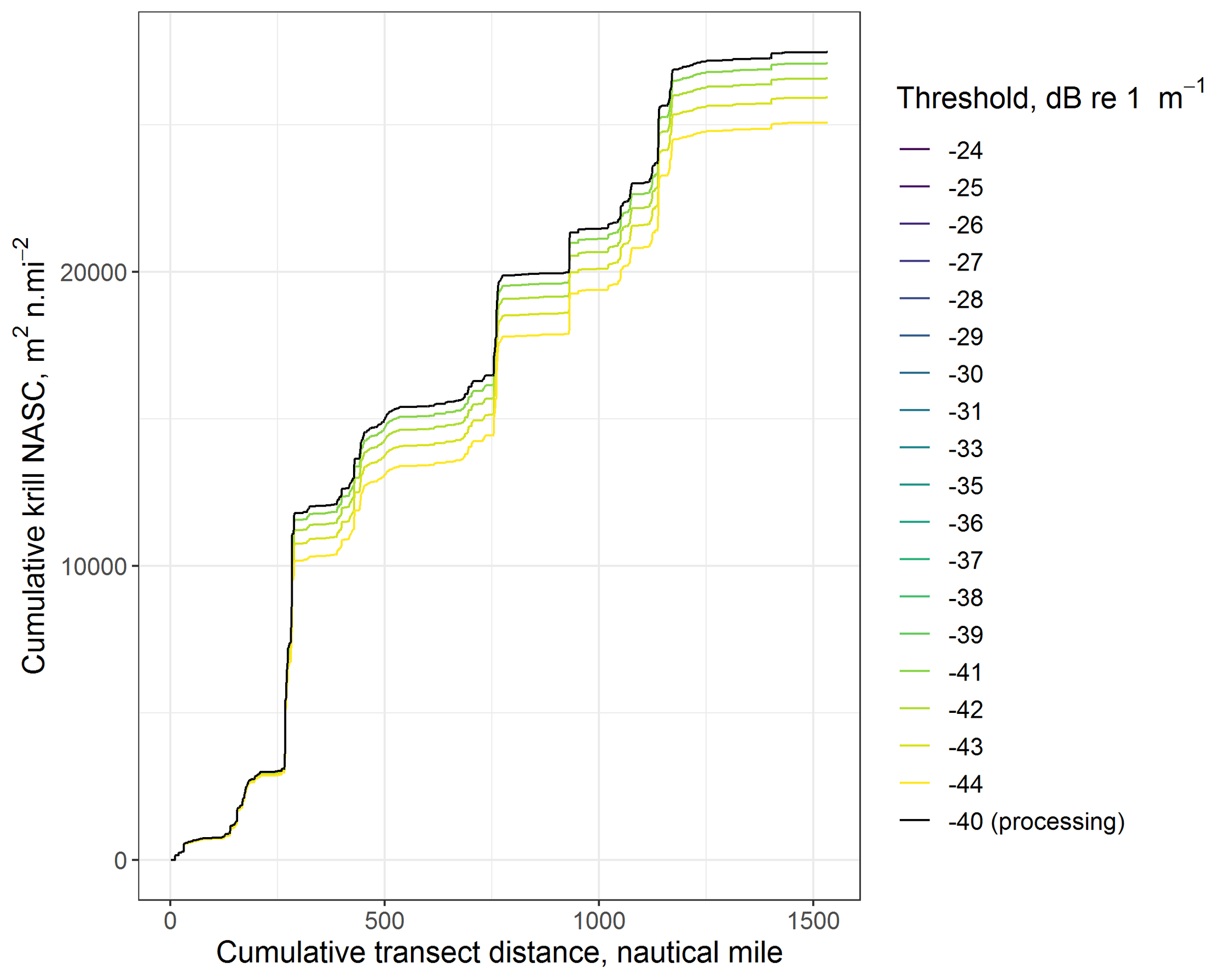

Supplement: S1 Fig — The noise removal filter Sv threshold can remove low-volume, high-density, krill echoes. Following the procedure of [14] the effect of varying the noise removal filter Sv threshold was investigated. The TEMPO voyage Sv data was processed with a noise removal threshold of—40 dB re 1 m-1 (solid black line) which did not remove any krill echoes. Lower value thresholds (-41 to -44 dB re 1 m-1) removed krill echoes. (TIF) [file pone.0271078.s002.tif]
